# Supplementary material for: Peripheral Intravenous Therapy in Internal Medicine Department—Antibiotics and Other Drugs’ Consumption and Characteristics of Vascular Access Devices in 2-Year Observation Study
Source: Antibiotics (Basel). 2024 Jul 18;13(7):664. doi: 10.3390/antibiotics13070664 (PMC11274068; doi:10.3390/antibiotics13070664)
Supplement: Supplementary file 1 [file antibiotics-13-00664-s001.zip › antibiotics-3063575-supplementary.pdf]

antibiotics

MDPI

Supplementary Material

Procedure for inserting a peripheral intravenous catheter (PIVC), Center of Pulmonology and Thoracic Surgery in Bystra, Bystra, Poland

The procedure for insertion of a peripheral intravenous catheter requires aseptic conditions using hand hygiene, disposable gloves, and disinfection of the cannula insertion site. Each PIVC secured with dressing must be evaluated twice a day for signs of vascular inflammation. The dressing was changed only if it is soiled, peeled off or damaged, and routine replacement of the catheter should not be performed if it was functioning properly and there were no signs of vasculitis. In situations where there was a suspicion of catheter related bloodstream infection (deterioration of patient's condition, fever, increased inflammatory parameters, local signs of infections), blood taken directly from the peripheral catheter and blood were taken from the peripheral vein elsewhere had to be secured for microbiological examination. After removal of the suspected PIVC, its tip cut off with sterile scissors should also be subjected to microbiological evaluation. Any intravenous administration of drugs had to be performed aseptically and using disposable equipment. After each medicine administration or completion of the infusion, the catheter had to be flushed with 3-5 ml of 0. 9% sodium chloride solution and sealed with a sterile plug. The nurse taking care of the patient was responsible for the proper care of the vascular line. The proper application of the procedure was supervised by the head of the unit and the infection control team.

Disclaimer/Publisher's Note: The statements, opinions and data contained in all publications are solely those of the individual author(s) and contributor(s) and not of MDPI and/or the editor(s). MDPI and/or the editor(s) disclaim responsibility for any injury to people or property resulting from any ideas, methods, instructions or products referred to in the content.
